# Prediction of CIAPIN1 (Cytokine-Induced Apoptosis Inhibitor 1) Signaling Pathway and Its Role in Cholangiocarcinoma Metastasis

**DOI:** 10.3390/jcm11133826

**Published:** 2022-07-01

**Authors:** Son Dinh An Truong, Molin Wongwattanakul, Tanakorn Proungvitaya, Temduang Limpaiboon, Sittiruk Roytrakul, Daraporn Chua-on, Doungdean Tummanatsakun, Siriporn Proungvitaya

**Affiliations:** 1 Centre of Research and Development of Medical Diagnostic Laboratories, Faculty of Associated Medical Sciences, Khon Kaen University, Khon Kaen 40002, Thailand; sontda@kkumail.com (S.D.A.T.); moliwo@kku.ac.th (M.W.); tanakorn@kku.ac.th (T.P.); temduang@kku.ac.th (T.L.); chuaondaraporn@kkumail.com (D.C.-o.); pui_ddlab41@hotmail.com (D.T.); 2 Faculty of Medical Laboratory Science, Danang University of Medical Technology and Pharmacy, Danang 550000, Vietnam; 3 Center for Innovation and Standard for Medical Technology and Physical Therapy, Faculty of Associated Medical Sciences, Khon Kaen University, Khon Kaen 40002, Thailand; 4 Cholangiocarcinoma Research Institute, Faculty of Medicine, Khon Kaen University, Khon Kaen 40002, Thailand; 5 National Center for Genetic Engineering and Biotechnology, National Science and Technology Development Agency, Pathum Thani 12120, Thailand; sittiruk@biotec.or.th

**Keywords:** cholangiocarcinoma, CIAPIN1, prognosis, siRNA, proteomics, metastasis, signaling pathway

## Abstract

Cholangiocarcinoma (CCA), a malignancy of the biliary epithelium, can arise at any point in the biliary system. We previously reported that CIAPIN1 is detectable in the sera and that its overexpression was associated with poor prognosis and metastasis of CCA patients. In this study, we investigated further its expression in CCA tissues, biological functions, and related signaling pathways in CCA cells. First, we examined CIAPIN1 expression in CCA tissues of 39 CCA patients using immunohistochemistry (IHC). Then, CIAPIN1-related proteins expressed in CCA cells were identified using RNA interference (siRNA) and liquid chromatography–mass spectrometry (LC–MS/MS). To predict the functions and signaling pathways of CIAPIN1 in CCA cells, the identified proteins were analyzed using bioinformatics tools. Then, to validate the biological functions of CIAPIN1 in the CCA cell line, transwell migration/invasion assays were used. CIAPIN1 was overexpressed in CCA tissues compared with adjacent noncancerous tissues. Its overexpression was correlated with lymph node metastasis. Bioinformatic analyses predicted that CIAPIN1 is connected to the TGF-β/SMADs signaling pathway via nitric oxide synthase 1 (NOS1) and is involved in the metastasis of CCA cells. In fact, cell migration and invasion activities of the KKU-100 CCA cell line were significantly suppressed by CIAPIN1 gene silencing. Our results unravel its novel function and potential signaling pathway in metastasis of CCA cells. CIAPIN1 can be a poor prognostic factor and can be a promising target molecule for CCA chemotherapy.

## 1. Introduction

Cholangiocarcinoma (CCA) is highly endemic in Northeast Thailand in relation to the high incidence of infection with the carcinogenic liver fluke, *Opisthorchis viverrini* (OV), which is the significant risk factor for CCA [1]. CCA is usually asymptomatic in the early stage and often diagnosed in the advanced stage, which highly compromises therapeutic options, resulting in a dismal prognosis [2]. In addition, patient outcome is poor due to metastasis [3]. Currently, combination chemotherapy with gemcitabine and cisplatin is the standard treatment for advanced or metastatic CCA patients. However, chemotherapy responses typically are limited and median overall survival is dismal (less than 12 months) [4]. Therefore, the identification of novel target molecules for CCA therapy is still required. For that, a better understanding of the critical signaling pathways and genetic alterations in CCA is necessary. There are certain mitochondrial features that are common in many cancers including tumor metastasis [5]. In the previous study of our group, Chua-On et al. identified a total of 281 mitochondrial proteins, 105 of which were overexpressed in the cancerous tissue of CCA compared with adjacent non-cancerous tissues [6]. Among them, cytokine-induced apoptosis inhibitor 1 (CIAPIN1) was identified as a potential prognostic biomarker for CCA [7]. In particular, the serum CIAPIN1 level was significantly higher in CCA patients with lymph node metastasis than those without metastasis [7].

The CIAPIN1 protein is encoded by the *CIAPIN1* gene, is accumulated in the nucleolus, and localized in the cytoplasm, nucleus, and mitochondria [6,8]. CIAPIN1, also known as anamorsin, is an anti-apoptotic molecule that has no sequence similarities to a series of apoptosis-associated molecules such as Bcl-2 or caspase family members. It is a key mediator of RAS signaling pathways and mediates the maintenance of hematopoiesis in the fetal liver [9]. CIAPIN1 is physiologically expressed in many tissues, especially self-renewing/proliferative tissues. It plays a biological role in many cancers as a candidate indicator for diagnosis, prognosis, and therapeutic target in various human cancers [10]. Although the expression level of CIAPIN1 has been found to relate to an oncogene or tumor suppressor in many different solid tumors [10], its expression, biological roles, and its mechanism in CCA have not been investigated. Remarkably, the involvement of CIAPIN1 in tumor metastasis mechanisms is still unknown. This study aimed to examine the expression of CIAPIN1 in CCA tumor tissue specimens and evaluate its prognostic value. Furthermore, to elucidate the biological role of CIAPIN1 in CCA cells, we attempted to prove the possible signal transduction pathways of CIAPIN1 and the associations of CIAPIN1-related proteins with chemotherapy drugs. The results demonstrated that CIAPIN1 might be a therapeutic target molecule for the prevention of CCA metastasis.

## 2. Materials and Methods

### 2.1. Patients and Specimens

The formalin-fixed, paraffin-embedded (FFPE) pathologically confirmed CCA tumor tissue specimens from 39 patients were provided by the Cholangiocarcinoma Research Institute, Faculty of Medicine, Khon Kaen University (CARI, KKU), Thailand. The patients underwent surgical operations at the Srinagarind Hospital, KKU, between 2010 and 2012. According to the reports from pathologists, lymph node metastasis was positive in 26 of 39 patients, whereas it was negative in 13 patients. The tissues used in this study were all leftover specimens obtained during the surgical treatment. Written informed consent for the use of leftover specimens for research purposes was obtained from the attending physicians before surgery. The original documents were kept in CARI, KKU. This series of experiments has been approved (HE631387) by the Ethics Committee of KKU, Thailand, and all specimens were anonymously coded according to the guidelines.

### 2.2. CCA Cell Lines

The three human CCA cell lines, KKU-M055 (JCRB1551), KKU-100 (JCRB1568) [11], and KKU-M213A, which was renamed from KKU-213 (JCRB1557) [12], and an immortalized cholangiocyte cell line, MMNK1 (JCRB1554) [13] were obtained from the Japanese Collection of Research Bioresources Cell Bank (Tokyo, Japan) and were kindly provided by CARI, KKU. The cell lines were cultured and subcultured as described previously [14]. All cell lines in this study were confirmed mycoplasma-free by specific PCR.

### 2.3. Immunohistochemistry and Evaluation of CIAPIN1 Expression in CCA Tissues

The IHC was conducted according to the previously reported procedures [15]. The rabbit polyclonal antibody against human CIAPIN1 (Cat#orb377996, Biorbyt, Cambridge, UK) was used for IHC staining at 1:150 dilution.

### 2.4. Western Blot Analysis

The Western blot analysis was performed for the detection of CIAPIN1 protein in CCA cell lysate as described previously [7]. Briefly, cells were lysed with lysis buffer (Cat#9806, RIPA, Cell Signaling Technologies, Danvers, MA, USA). Protein amounts were determined using the Bradford assay (Bio-Rad, Hercules, CA, USA). Twenty-five micrograms of cell lysate were dissolved in a sample buffer for Western blotting. The rabbit polyclonal antibody against human CIAPIN1 (Cat#orb377996, Biorbyt, Cambridge, UK) was used at 1:1000 dilution for Western blot analysis in this study. β-actin antibody (Cat#ab227387, Abcam, Cambridge, UK) was used for detecting a loading control.

### 2.5. Transient Silencing of CIAPIN1 Gene Using siRNA

Since CIAPIN1 expression in the cell lysates of KKU-100 and KKU-213A cells was higher than that of the other CCA cell lines, we selected these cell lines for the CIAPIN1 gene silencing experiments. In brief, for the CIAPIN1 gene silencing using a siRNA technique, the cells (4.5 × 10^5^ cells/well) were seeded in a 6-well plate and cultured overnight before being transfected with 75 pM of siCIAPIN1 (Cat#sc-60168, Santa Cruz Biotechnology, Inc., Dallas, TX, USA), while scrambled siRNA (Cat#AM4611, Invitrogen, Carlsbad, CA, USA) was used as a negative control. Transfection was carried out using Lipofectamine 3000 (Invitrogen, Carlsbad, CA, USA) according to the manufacturer’s instructions. After 6 h of transfection, the culture medium was replaced with a complete medium, and the plates were incubated at 37 °C for 24 h. To check the suppression of protein expression, the cells were harvested in lysis buffer and incubated at 4 °C for 10 min. The cell lysate was centrifuged at 20,000× *g* (4 °C) for 30 min. The level of CIAPIN1 protein was determined using Western blot analysis with β-actin as a loading control. In addition, CIAPIN1-silenced and control scrambled cells were tested for migration and invasion assay in vitro as given below.

### 2.6. Sample Preparation for Tryptic Digestion

After transfection of siRNA or scramble RNA, cell lysates of CIAPIN1 gene-silenced and scramble-treated control cells were prepared and their protein content was determined by Lowry assay using BSA as a protein standard. In-gel digestion was performed using an in-house method developed by the Functional Proteomics Technology Laboratory, National Center for Genetic Engineering and Biotechnology, Thailand. Briefly, 4 µg of total proteins were directly incorporated into a 12.5% polyacrylamide and congealed in a microtube, reduced disulfide bonds using 5 mM dithiothreitol (DTT) in 10 mM AMBIC at 60 °C for 1 h, and alkylated sulfhydryl groups using 15 mM iodoacetamide (IAA) in 10 mM AMBIC at RT for 45 min in the dark. For digestion, samples were mixed with 50 ng/µL of sequencing grade trypsin (1:20 ratio) (Promega, Walldorf, Germany) and incubated at 37 °C overnight. Prior to LC–MS/MS analysis, the digested samples were dried and protonated with 0.1% formic acid before injection into LC–MS/MS. The LC–MS/MS data analysis and protein identification were performed as described previously [16]. The LC–MS analysis of each sample was done in triplicate.

### 2.7. Selection of CIAPIN1-Related Proteins Using Jvenn

The effects of CIAPIN1 silencing on protein expression patterns of cell lysates were determined using the mass spectrometry data sets of siRNA and scramble-treated cells. Overlapping and unique proteins were identified using jvenn software which is an integrative web-tool for comparing lists with Venn Diagrams and accessed on 5 November 2020 (http://bioinfo.genotoul.fr/jvenn) [17]. To speculate on the active role(s) of CIAPIN1 in CCA and its possible signaling pathway, proteins that were uniquely expressed in scramble-treated, but not in siRNA-treated KKU-213A and KKU-100 cells, were selected as the candidate protein group.

### 2.8. Protein–Protein Interaction Analysis for Prediction of CIAPIN1 Signaling Pathway and Relationship with Chemotherapeutic Drugs

The potential interaction of the identified proteins was analyzed using the search tool STITCH version 5.0 (http://stitch.embl.de/, accessed on 15 November 2020). In brief, 150 gene names of related proteins were put into a box of multiple name items to predict the CIAPIN1 signaling pathway. Meanwhile, chemotherapeutic drugs (cisplatin, 5-fluorouracil, gemcitabine, doxorubicin) were added for exploring the interaction between CIAPIN1 and chemotherapeutic drugs. Then “Homo sapiens” was selected as the organism. In order to obtain an optimal network configuration, a default intermediate confidence score of >0.4 was used; and our set of protein and the first spheres of interaction were varied in the range of 10 nodes. To interpret the interaction view, strong associations are represented by the thicker lines, weak associations by thin lines, protein–protein interactions in grey, chemical–protein interactions in green, and interactions between chemicals in red lines.

### 2.9. KEGG Pathway Enrichment Analysis to Predict CIAPIN1 Signaling Pathway

DAVID was used to identify significant groups of genes and pathways that are related to the data set used in this study. All 150 CIAPIN1-related proteins were imported into the DAVID Bioinformatics resource version 6.8 (https://david.ncifcrf.gov/, accessed on 15 November 2020) for pathway annotation analysis. Generated files of KEGG pathway analysis were obtained. The EASE score threshold (maximum probability) = 0.1, as a default (*p*-value), was selected.

### 2.10. Correlation between CIAPIN1 and Related Proteins in the Signaling Pathway

To verify the correlation between CIAPIN1 and related proteins in the signaling pathways, we used mRNA expression data of CCA and a normal tumor from the open-access internet database of Gene Expression Profiling Interactive Analysis (GEPIA 2, http://gepia2.cancer-pku.cn/, Peking University, Beijing, China, accessed on 15 November 2020) [18]. The correlation of mRNA expression levels between CIAPIN1 and related proteins in the signaling pathways was determined using the pairwise analysis of the Spearman statistic tool. A *p*-value < 0.05 was considered for the statistical significance.

### 2.11. Molecular Docking Verification of CIAPIN1 Binding to Its Related Proteins and Chemotherapeutic Drugs

To identify potential interactions between a given protein and a ligand, CB-Dock was used (http://cao.labshare.cn/cb-dock/ accessed on 16 May 2021). We obtained the three-dimensional (3D) X-ray crystal structure of human CIAPIN1 (PDB ID: 4M7R, solution 1.8 Å), NOS1 (PDB ID: 6CID, solution 1.75 Å), and SMAD2 (PDB ID: 6YIA, solution 1.30 Å) from the Protein Data Bank (PDB; accessed on 16 May 2021). The structure of doxorubicin (Compound CID: 31703) was obtained from PubChem (https://pubchem.ncbi.nlm.nih.gov/). In brief, a protein file in the PDB format and a ligand file in the MOL2, MOL, or SDF, were derived from the protein database (http://www.rcsb.org) to input into CB-Dock. To prepare the ligand files, Open Babel was used to convert NOS1 and SMAD2 to SDF format [19]. Next, using CB-Dock, we predicted cavities of the protein and calculated the centers and sizes of the top N (N = 5 by default) cavities. The final results are displayed after the computation of N rounds, including binding scores, cavity sizes, and docking parameters of the predicted binding modes. The 3D structures of the highest binding modes on the web page were selected and presented. The lower the Vina scores are, the more stable the ligand binding to the receptor [20], the reference of Vina scores as a cut-off was less than −5.6 kcal/mol [21].

### 2.12. Transwell Cell Migration and Invasion Assay

The effect of CIAPIN1-silencing on CCA cell motility was determined using a transwell 24-well plate. For the migration assay, the chamber was equipped with the membrane filter of 8 µm pore size (Corning, Kennebunk, ME, USA), and for the invasion assay, the chamber was equipped with the Matrigel-coated membrane (Corning, Kennebunk, ME, USA). CIAPIN1-silenced and scramble-treated KKU-100 cells were inoculated into the upper chamber with a cell density of 4 × 10^4^ cells/200 µL of serum-free medium. The lower chamber was filled with 600 µL of complete medium. After incubation for 48 h, migrated cells on the lower surface of the membrane were fixed with 100% methanol and stained with 2% crystal violet in 2% ethanol for 15 min. They were then washed in 1xTBS 5 times. The migrated/invaded cells were counted as the number of nuclei under an inverted microscope fitted with an objective lens of 10×. Six randomized fields for each membrane filter were counted [22].

### 2.13. Statistical Analysis

The data are presented as the median ± quartile deviation or the mean ± standard deviation (with the minimum to the maximum range). The difference in the values between two independent sample groups was estimated using the Mann–Whitney U test. The association between serum CIAPIN1 levels and patients’ clinicopathological parameters was analyzed using Fisher’s exact test. X-tile software version 3.6.1 [23] was used to find out the optimal cut-off values of CIAPIN1 expression in tissues in relation to the prognosis of CCA. This cut-off was used to dichotomize its relative intensity as low and high levels and their correlation with clinicopathological parameters including survival time was determined. The Cox proportional hazards regression model was used for univariate and multivariate analysis. *p* < 0.05 was considered statistically significant. IBM SPSS v.26 software (IBM Corp., Armonk, NY, USA) was used for statistical analyses.

## 3. Results

### 3.1. Immunohistochemical Detection of CIAPIN1 Protein in CCA Tissues

**Figure 1:**
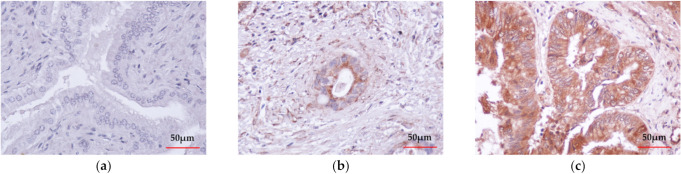

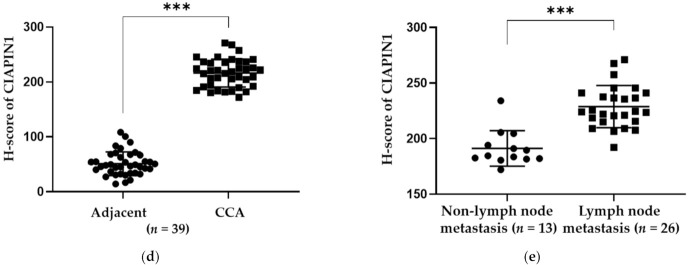
Immunohistochemical detection and the representative images of CIAPIN1 in CCA tissues (taken at a magnification of 400×). (**a**) The negative control of CCA tissue without primary antibody followed by secondary antibody. (**b**) Weak CIAPIN1 staining of the normal monolayer of cholangiocytes in normal tissues adjacent to CCA tissue. (**c**) Strong cytoplasmic staining in CCA cells. (**d**) The expression of CIAPIN1 in the cancerous region was higher than in the adjacent region with statistical significance. (**e**) CIAPIN1 expression in cancerous regions of the lymph node (LN) metastasis group was higher than the non-metastasis group with statistical significance. Data were presented as mean ± SD; Mann–Whitney U test was used; *** statistically significant with *p* < 0.0001.

The expression of CIAPIN1 in CCA was investigated immunohistochemically using 39 CCA tissues. The results showed that CIAPIN1 was stained predominantly in the cytoplasm of both cancerous and normal cells (Figure 1b,c). Figure 1a is a negative control staining of CCA tissue without primary antibodies. Using the H-score system, CIAPIN1 was highly (*p* < 0.0001) expressed in CCA (mean ± SD of H-score = 216 ± 25), compared with normal bile ducts in the CCA adjacent areas (mean ± SD of H-score = 51 ± 21) (Figure 1d). In addition, comparing patients with and without lymph node metastasis, our data showed that CIAPIN1 expression in CCA of the patients in the lymph node metastasis group was significantly higher than that of the non-metastasis group (*p* < 0.0001, Figure 1e).

### 3.2. Association between CIAPIN1 Expression in CCA Tissues and Clinicopathological Features of Patients

We analyzed the relationship between CIAPIN1 expression in cancerous tissue and the clinicopathological characteristics of CCA patients. At first, using X-tile software, the optimal cut-off value of CIAPIN1 H-score to divide CCA patients into high and low CIAPIN1 expression groups was determined to be 207.5 (Figure S1). Accordingly, 15 of 39 CCA patients were in the low expression group and 24 in the high expression group. The patients’ characteristics such as age, gender, tumor size, the histopathological grade with non-papillary and papillary, vascular invasion, and lymph node metastasis were summarized in Table 1. As can be seen, high CIAPIN1 expression was significantly correlated with lymph node metastasis (*p* = 0.010), intraductal invasion (*p* = 0.048), and overall survival time (*p* = 0.013).

### 3.3. High Expression of CIAPIN1 Is Associated with Poor Prognosis of CCA Patients

**Figure 2:**
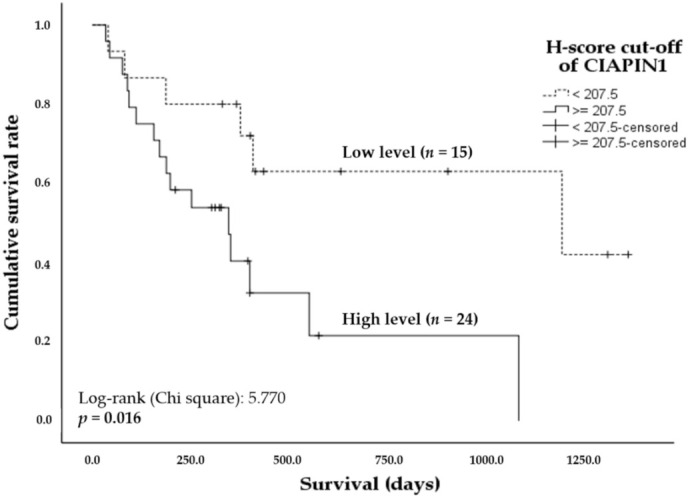
Kaplan–Meier plots showing the comparison of overall survival of CCA patients according to CIAPIN1 expression. The curves represent the overall survival time of CCA patients having high (solid line) and low (dashed line) H-score levels. A significant difference of the survival time was observed between high and low H-score CIAPIN1 level groups by log-rank test and *p* < 0.05.

A log-rank test with Kaplan–Meier estimates was adopted to determine whether CIAPIN1 expression in CCA tissues can be a prognostic factor for the survival of CCA patients. The overall survival analysis using the Kaplan–Meier method revealed the mean (95%CI) survival time of CCA patients with low and high CIAPIN1 expression in CCA tissues was 799 days (530–1067) and 462 days (258–667), respectively. In addition, higher CIAPIN1 expression was associated with shorter survival time (Figure 2, *p* = 0.016). The Cox proportional hazards model analysis was conducted to examine the effect of the covariates, including age, sex, invasion, lymph node metastasis, histology grading, and the tissue CIAPIN1 expression level, showing that only CIAPIN1 is an independent factor for poor prognosis of CCA (HR = 4.01, 95% CI: 1.131–14.195; *p* = 0.031, Table 2). Then, we hypothesized that CIAPIN1 might affect cell behavior such as metastasis of CCA. To substantiate this hypothesis, we carried out CIAPIN1 gene silencing and bioinformatic analyses using CCA cell lines.

### 3.4. Expression of CIAPIN1 in CCA Cell Lines

**Figure 3:**
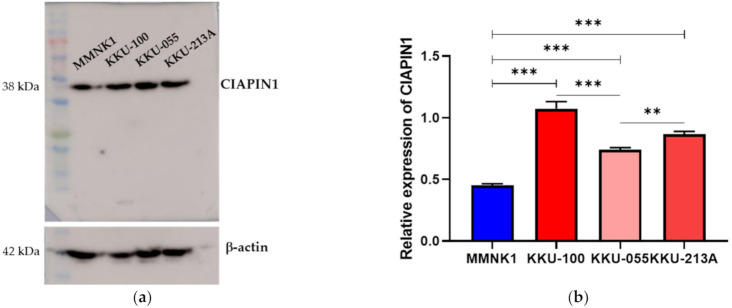
Expressions of CIAPIN1 in CCA cell lines. (**a**) The protein levels of CIAPIN1 were determined by western blotting (WB) in CCA cell lines. (**b**) Relative expression of CIAPIN1 in CCA cell lines was examined by WB and the relative expression was normalized to β-actin, which is a loading control. The data were presented as the mean ± SD from triplicate independent experiments (** *p* < 0.01; *** *p* < 0.001).

We first compared the expression of CIAPIN1 in three CCA cell lines and an immortal biliary epithelial cell line, MMNK1. As shown in Figure 3, the level of CIAPIN1 was remarkably variable among all four cell lines (KKU-100, KKU-213A, KKU-055, and MMNK1). From this result, we selected KKU-M213 and KKU-100 for CIAPIN1 gene silencing experiments.

### 3.5. Effects of CIAPIN1 Gene Silencing of CCA Cell Lines

**Figure 4:**
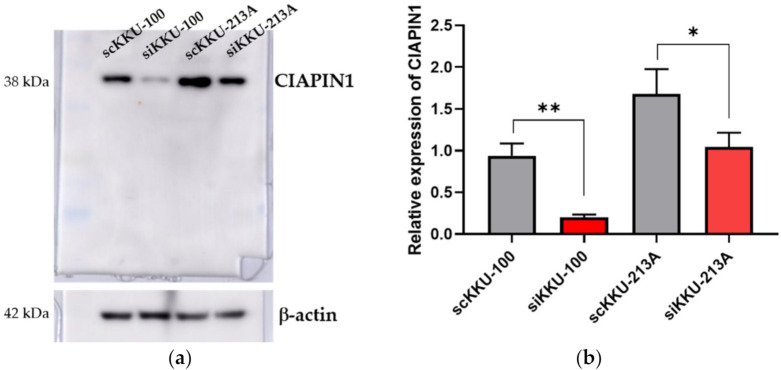
The effects of CIAPIN1 gene silencing on the CCA cell lines. (**a**) Western blot analysis showing suppressed CIAPIN1 protein after gene silencing. (**b**) Western blot examined the relative expression of CIAPIN1 in CCA cell lines after gene silencing and normalization to β-actin expression. β-actin was used as a control for loading protein. scKKU-100 and scKKU213A are scramble-treated (without siRNA treatment) cells; siKKU-100 and siKKU213A are CIAPIN1-silenced-treated cells. The data were presented as the mean ± SD from triplicate independent experiments (* *p* < 0.05; ** *p* < 0.01).

To investigate the biological roles of CIAPIN1 in CCA cells, the effects of CIAPIN1 gene suppression were examined using siRNA on both KKU-M213A and KKU-100 cells. As shown in Figure 4, CIAPIN1 expression of both CCA cell lines was transiently suppressed by siRNA at 24 h after transfection. Western blot analysis showed that the expression of CIAPIN1 was successfully suppressed in both KKU-M213A and KKU-100 cells with dominant suppression in KKU-100. Then, to predict biological function and possible signaling pathways of CIAPIN1, proteomic analysis of gene-silenced and scramble KKU-213A and KKU-100 cells at 24 h was performed using mass spectrometry.

### 3.6. Protein Expression Patterns of CIAPIN1 Gene-Silenced and Scramble-Treated KKU-213A and KKU-100 Cell Lines

**Figure 5:**
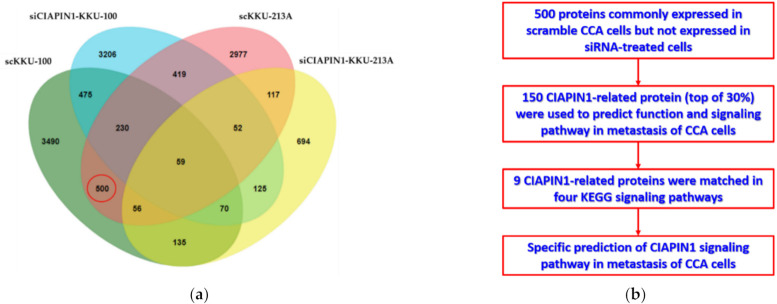
Identification of CIAPIN1-related proteins. (**a**) Venn diagram presents the number of proteins in each sample and the degree of individual overlap among CIAPIN1 gene-silenced and scramble-treated KKU-213A and KKU-100 cells. The red circle illustrates the number of candidate proteins as CIAPIN1-related proteins. (**b**) Flowchart of selection CIAPIN1-related proteins and prediction of the signaling pathway in metastasis of CCA cells.

Then, to predict possible signaling pathways of CIAPIN1, proteomic analysis of gene-silenced and scramble-treated KKU-213A and KKU-100 cells was performed at 24 h. A total of 16,357 proteins were isolated from the cell lysates of CIAPIN1 gene-silenced and scramble-treated KKU-213A and KKU-100 cell lines. Then, using UniProt, 3752 proteins without gene names were excluded. Using jvenn software, we identified 4410 proteins from scramble-treated KKU-213A, 1308 from siRNA-treated KKU-213A, 5015 from scramble-treated KKU-100, and 4636 from siRNA-treated KKU-100 (Figure 5a). Among them, we identified 500 proteins that were commonly expressed in scramble KKU-213A and KKU-100 cells but not expressed in siRNA-treated cells (Table S1). According to the MS intensity of each protein, the top 30% of 500 proteins were selected for further analysis to predict functions and signaling pathways of CIAPIN1 in CCA cells (Figure 5b).

### 3.7. Construction of Protein–Protein Interaction Network of CIAPIN1 and its Related Proteins

**Figure 6:**
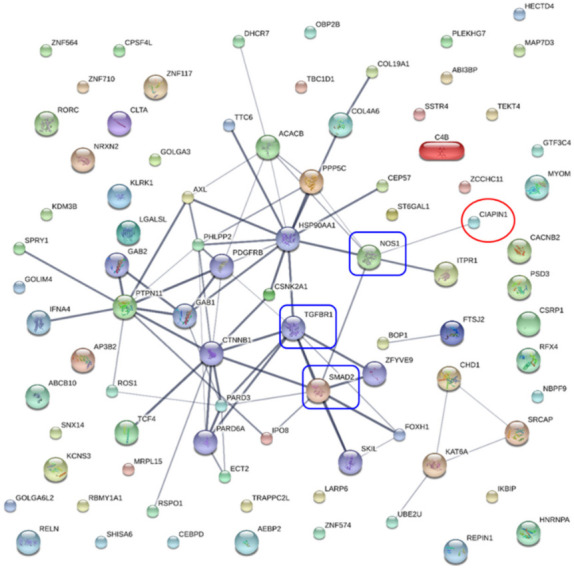
Protein interaction networks of CIAPIN1 and related proteins of scramble-treated CCA cells. CIAPIN1 interacted directly with nitric oxide synthase 1 (NOS1), and indirectly with mothers against decapentaplegic homolog 2 (SMAD2) and transforming growth factor beta receptor 1 (TGFβR1). Thicker lines represent strong associations. Thin lines represent weak associations.

To identify potential CIAPIN1 signaling pathways and functions, the protein–protein interaction (PPI) network was constructed by importing 150 CIAPIN1-related proteins selected above to the STITCH version 5. As shown in Figure 6, the PPI network indicated several possible signaling pathways. Among them, we speculated that the signaling pathway of CIAPIN1 might be mediated via nitric oxide synthase 1 (NOS1), to TGFβR1/SMAD2 system. Then, the KEGG pathway enrichment analysis was performed by running DAVID version 6.8.

### 3.8. Prediction of CIAPIN1 Signaling Pathways in CCA Cells

To elucidate possible functions and signaling pathways of CIAPIN1 in CCA cells, KEGG pathway enrichment analysis was performed by importing 150 CIAPIN1-related proteins using DAVID version 6.8. The results show that nine CIAPIN1-related proteins including ERBB3 (Receptor tyrosine-protein kinase erbB-3), ITPR1 (Inositol 1,4,5-trisphosphate receptor type 1), CACNA1A (Voltage-dependent P/Q-type calcium channel subunit alpha-1A), ADRA1D (Alpha-1D adrenergic receptor), NOS1, ADRA1A (Alpha-1A adrenergic receptor), SMAD2, CSNK2A1 (Casein kinase II subunit alpha), and PARD3 (Partitioning defective 3 homolog) were involved in four KEGG signaling pathways with variable combinations (Table 3). In terms of CCA biology, both the calcium signaling pathway and the adherens junction signaling pathway are assumed to be involved in tumor metastasis mechanisms. Moreover, these nine matching proteins were related to cell migration, invasion, and metastasis of tumor cells. Their names, abbreviations, and synonyms were used for further analysis.

**Figure 7:**
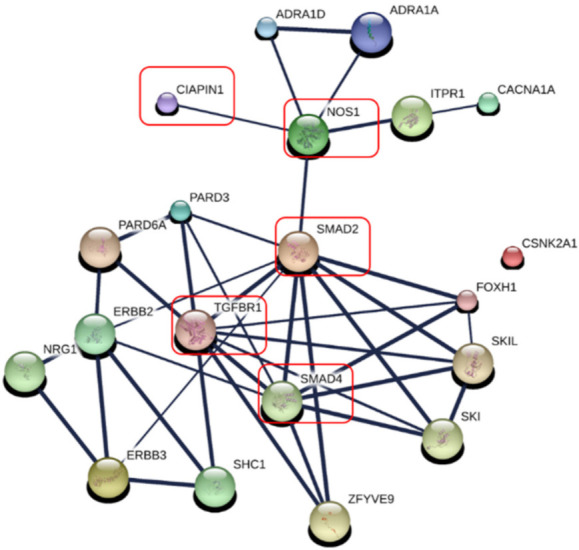
Specific prediction of CIAPIN 1 signaling pathway in metastasis of CCA cells. CIAPIN1 was predicted to be involved in the transforming growth factor beta (TGF-β) and SMADs signaling pathway through NOS1 in the metastasis process of CCA cells (red rectangle). Thicker lines represent strong associations. Thin lines represent weak associations.

To predict the potential signaling pathways of CIAPIN1 in CCA cells, these nine CIAPIN1-related proteins that appeared in calcium signaling and adherence junction signaling pathways were imported only to STITCH. PPI analysis indicated that CIAPIN1 interacts with this set of nine proteins (ERBB3, ITPR1, CACNA1A, ADRA1D, ADRA1A, SMAD2, CSNK2A1, and PARD3) through NOS1. Especially, it reacted to SMAD2, SMAD4, and TGFBR1 via NOS1 (Figure 7), which was accordant to the PPI network of 150 CIAPIN1-related proteins as shown in Figure 7. Thus, we speculate that CIAPIN1 was involved in the TGF-β/SMADs signaling pathway via NOS1 in metastasis of CCA cells.

### 3.9. Correlation between CIAPIN1 and its Related Proteins in Signaling Pathway

**Figure 8:**
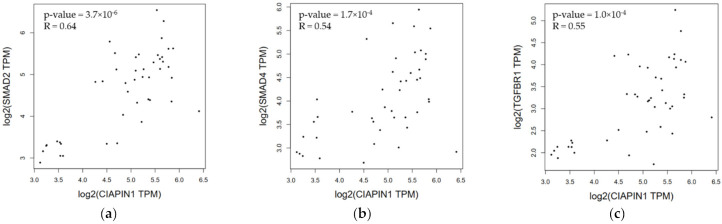
Correlation of mRNA expression of pair genes. (**a**) CIAPIN1 and SMAD2; (**b**) CIAPIN1 and SMAD4; and (**c**) CIAPIN1 and TGFβR1 in CCA and normal tumor. Data were retrieved from GEPIA2 tool as log2 of transcript per million (TPM) using the Spearman correlation analysis, *p* < 0.05.

Since, via NOS1, CIAPIN1 might be involved in TGF-β/SMADs signaling pathway in the metastasis process of CCA cells (Figure 7). The Spearman correlation analysis tool on the open-access internet database of GEPIA2 was used to verify the relationship between CIAPIN1 and related proteins in CCA and normal tissue (Figure 8). The scatter plots showed that the mRNA level of CIAPIN1 significantly correlated with that of SMAD2, SMAD4, and TGFβR1 which suggested strong and moderate positive association (R = 0.64, *p* < 0.0001; R = 0.54, *p* < 0.0001; and R = 0.55, *p* < 0.0001; respectively).

### 3.10. Molecular Docking of Protein–Ligand between CIAPIN1 and Each Related Proteins in Signaling Pathway

**Figure 9:**
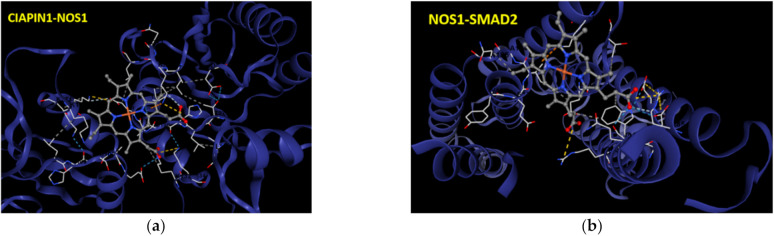
The interactive 3D viewer illustrating the highest Vina score of binding mode and representative positions of protein–ligand docking at their pocket binding sites. Blue cartoon ribbons represent (**a**) CIAPIN1 protein interface to the NOS1 (ball and stick) and (**b**) SMAD2 protein interface to the NOS1 (ball and stick). Interaction bond is presented by the dashed line.

To verify the protein–protein interaction of CIAPIN1 and its related proteins in the predicted signaling pathway, CB-Dock analysis was performed to see the binding geometries of the key interactions. According to PPI networks in Figure 6 and Figure 7, we selected the docking between CIAPIN1 and NOS1, and NOS1 and SMAD2. After docking analysis, their Vina scores were less than −5.6 kcal/mol which is the cut-off. The highest Vina score between CIAPIN1 and NOS1 was −7.6 kcal/mol and the largest cavity size was 7734 (Figure 9a), whereas those between NOS1 and SMAD2 were −6.6 kcal/mol Vina score, and 443 cavity size (Figure 9b). In accordance with the results of PPI analysis using STITCH, CB-Dock indicated strong binding of CIAPIN1 to NOS1 which interacts with SMAD2.

### 3.11. Effect of CIAPIN1 Gene Silencing on Cell Motilities of CCA Cell Line

**Figure 10:**
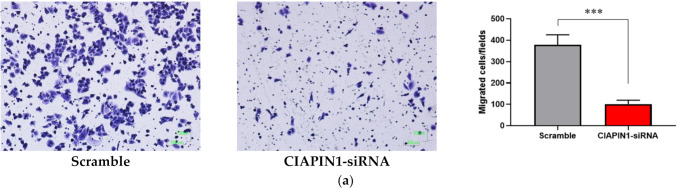

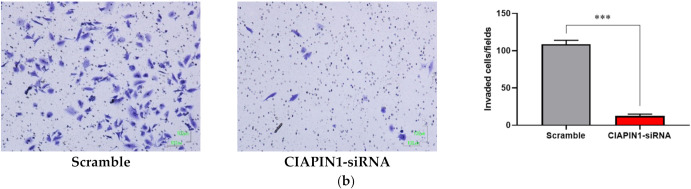
The effects of CIAPIN1 gene silencing on cell migration (**a**) and invasion (**b**) of KKU-100. The cells treated without siRNA-CIAPIN1 (scramble, SC) were used as controls. The data were presented as the mean ± SD from triplicate independent experiments (*** *p* < 0.0001).

Since CIAPIN1 was predicted to activate the TGF-β/SMADs signaling pathway via NOS1 in CCA metastasis, we investigated the role of CIAPIN1 in the migration and invasion of CCA cells. For this purpose, we selected the KKU-100 cell line because the CIAPIN1 protein expression level in the cell lysate was much higher than the other two cell lines (Figure 3). When the *CIAPIN1* gene of KKU-100 cells was silenced using siRNA, both cell migration (Figure 10a) and invasion (Figure 10b) were significantly (*p* < 0.0001) suppressed.

### 3.12. The Correlation of CIAPIN1 and its Related Proteins to Chemotherapeutic Drugs

**Figure 11:**
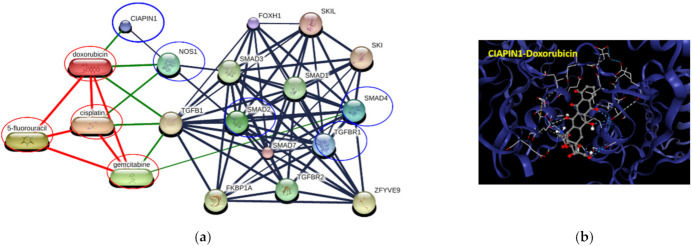
Involvement of CIAPIN1 and related proteins in networks of protein–chemotherapeutic drug interaction. (**a**) The interactive 3D viewer illustrates the highest Vina score of binding mode and represents positions of protein–ligand docking at their pocket binding sites. Blue cartoon ribbons represent the CIAPIN1 protein interface to the doxorubicin ligand (ball and stick). The interaction bond is presented by the dashed line. (**b**) Protein–chemical interaction of CIAPIN1 (bold blue circle), related proteins (blue circles), and chemotherapeutic drugs cisplatin, 5-fluorouracil, gemcitabine, and doxorubicin (red circles). Thicker lines represent strong associations. Thin lines represent weak associations.

To elucidate whether CIAPIN1 can be a molecular target for cancer chemotherapy, we predicted the networks of CIAPIN1 and related proteins in the signaling pathway and chemotherapeutic drugs using STITCH. The results showed that CIAPIN1 interacts directly with doxorubicin and indirectly with cisplatin via NOS1 (Figure 11a). In CB-Dock analysis, the highest Vina score between CIAPIN1 and doxorubicin was −8.4 kcal/mol with the largest cavity size of 7734 (Figure 11b). Thus, CIAPIN1 can strongly bind to doxorubicin. Moreover, PPI analysis revealed that CIAPIN1, NOS1, doxorubicin, and cisplatin interacted with the TGFβ/SMADs system (Figure 11a). Hence, CIAPIN1 might play a role in cancer drug resistance and be a molecular target for cancer chemotherapy.

## 4. Discussion

In oncogenesis research, the overexpression of CIAPIN1 has been reported in various solid tumors and is considered an oncogene or tumor suppressor in different tumor types. However, the expression and role of CIAPIN1 in CCA have remained unknown. Recently we have found that the serum CIAPIN1 level is associated with the prognosis of CCA patients [7]. In the present study, we investigated the expression of CIAPIN1 in human CCA tissues and CCA cell lines to elucidate the role of CIAPIN1 and its molecular mechanisms in CCA tumorigenesis/tumor progression.

Using immunohistochemical methods, we demonstrated that CIAPIN1 is overexpressed in the majority of CCA tissues (Figure 1). Our data were in accordance with the previous data of bioinformatic analysis of CIAPIN1 expression in CCA tissue compared with normal samples using the GEPIA2 database [7]. Furthermore, CIAPIN1 expression was higher in CCA tissues of patients with lymph node metastasis (Figure 1e). Accordant with CIAPIN1 expression in CCA tissues, the serum CIAPIN1 level in CCA patients with lymph node metastasis was higher than that without metastasis [7]. Overexpression of CIAPIN1 was reported in epithelial ovarian cancer [24] and metastatic ovarian serous carcinoma [25]. These data indicate that CIAPIN1 may play an oncogenic role in diverse cancers. Our data suggest that *CIAPIN1* might act as an oncogene to promote the malignant progression of CCA.

In this study, CIAPIN1 is an independent prognostic factor for CCA. In addition, upregulation of CIAPIN1 is related to lymph node metastasis. In our previous study, the serum CIAPIN1 levels was an independent unfavorable prognostic factor for patients with CCA [7]. Already, Lopes et al. reported that the expression analysis of CIAPIN1 may predict metastasis and poor prognosis of patients with gastric cancer [26]. Furthermore, the decrease in CIAPIN1 expression is significantly associated with a longer survival time of diffuse large B cell lymphoma [27], colorectal cancer [28], pancreatic cancer [29], and non-small-cell lung carcinoma [30]. A further study with large sample size is needed to unravel the correlation between lymph node metastasis and CIAPIN1 expression, and also to identify whether CIAPIN1 can be a precise and reproducible prognostic marker and metastasis indicator for CCA patients. Moreover, this approach should expand to other types of cancers.

In the present study, using a combination of gene silencing and mass spectrometry techniques followed by bioinformatic analysis revealed that CIAPIN1 and related proteins are involved in calcium signaling and adherens junction pathways, both of which are related to tumor metastasis [31,32]. Moreover, STITCH analysis combined with KEGG signaling pathway search revealed that, via NOS1, CIAPIN1 interacted with nine proteins related to cell migration, invasion, and metastasis of tumor cells (Figure 7). Thus, CIAPIN1 may play an important role in the metastatic process of CCA. To validate the role of CIAPIN1 in CCA metastasis, we examined the effects of CIAPIN1 gene silencing on cell migration and invasion of CCA cells. The results showed that cell motility was significantly suppressed after CIAPIN1 silencing (Figure 10). In contrast to our results, in the case of non-small-cell lung carcinoma (NSCLC), CIAPIN1 overexpression inhibited cell migration and invasion and suppressed the expression of MMPs and EMT-associated markers [30]. Thus, CIAPIN1 might have a contrary function among different cancers or even within CCA via different signal pathways. Further experiments are needed to validate possible multifunctional roles of CIAPIN1 in cancer, more CCA cell lines, and also other tumor cell lines should be used to explore the biological function of CIAPIN1 in various cancers.

**Figure 12:**
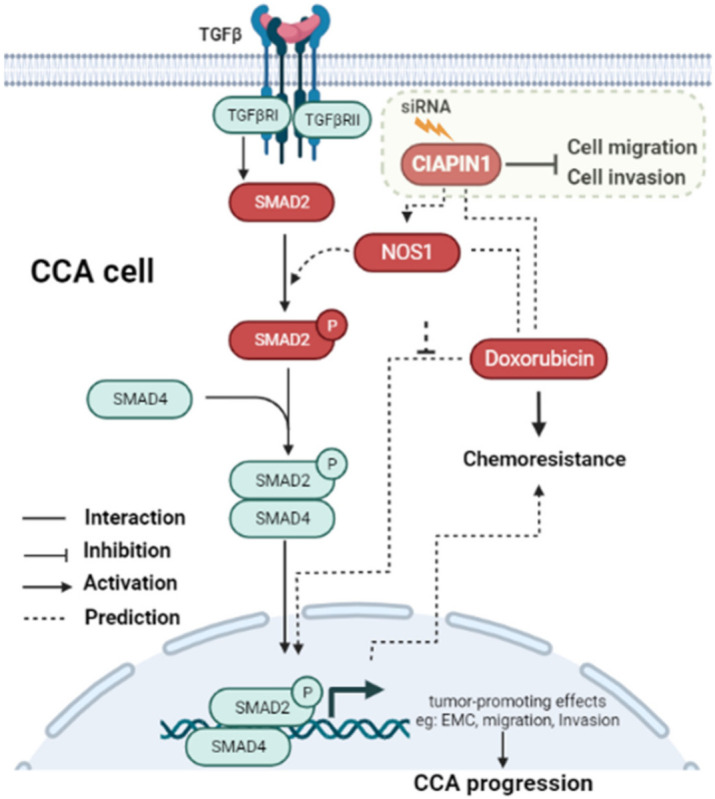
Schematic representation of the predicted signaling pathway of how CIAPIN1 promotes the metastasis and chemoresistance of CCA. CIAPIN1 was predicted to activate SMAD2 signal transduction and involve in the TGF-β signaling pathway via NOS1. The result indicated an increase in migration and invasion, which lead to metastasis in CCA. CIAPIN1 interacted directly with doxorubicin, led to promote chemoresistance function in CCA cells. The molecules in red rectangles were demonstrated in the present study.

In this study, PPI analysis illustrated that CIAPIN1 interacted with SMAD2, SMAD4, and TGFβR1 via NOS1 (Figure 7). In addition, the Spearman correlation analysis of GEPIA2 showed a positive correlation of the mRNA level of CIAPIN1 with that of SMAD2, SMAD4, and TGFβR1 (Figure 8). Moreover, NOS1 and SMAD2 were expressed only in scramble-treated CCA cells. Thus, we speculated that NOS1 and SMAD2 could be downstream of the CIAPIN1 signaling pathway. In fact, CB-Dock analysis revealed a strong binding between CIAPIN1 and NOS1, and also NOS1 and SMAD2 (Figure 9). Recently, Medvedev et al. demonstrated the direct interaction between NOS1 and SMAD2 [33], both of which are involved in TGF-β signaling [34,35], suggesting the involvement of TGF-β signaling downstream of CIAPIN1. In previous studies, NOS1 is overexpressed in various types of cancer, and its expression is associated with tumor progression. For instance, higher NOS1 expression promotes the proliferation and invasion of ovarian cancer [36]. Accordingly, nitric oxide (NO) is synthesized from L-Arginine and oxygen by a family of enzymes termed nitric oxide synthases, including NOS1, which regulates the TGF-β signaling of endothelial cells [34]. Moreover, TGF-β is one of the major signaling pathways that promote CCA progression. TGF-β induces EMT in CCA cell lines [37]. Interestingly, tumor invasion and migration are also mediated via the TGF-β/SMAD4 signaling pathway [38]. Taken together, we speculate that, via NOS1, CIAPIN1 activates the TGF-β/SMADs signaling pathway to augment tumor metastasis (Figure 12). Since all those signaling pathways are speculated and based on results of in silico analysis, further direct experiments are required to confirm the interaction between CIAPIN1 and NOS1, and the modulation of CIAPIN1 on up and downregulation of these target expressions through TGF-β/SMADs signaling pathway.

In terms of multidrug resistance, Wang et al. reported that overexpression of CIAPIN1 contributes to multidrug resistance (MDR) in breast cancer. CIAPIN1 gene silencing enhanced the sensitivity of tumor cells to doxorubicin in drug-resistant breast cancer xenografts in this nude mouse model [39]. Moreover, Lu et al. showed that, in the MCF7/ADM cell line of breast cancer, CIAPIN1 gene silencing by siRNA reduced the drug resistance against epirubicin, paclitaxel, and gemcitabine by regulating MDR1 and P53 expression [40]. However, the mechanism of its effects on the chemoresistance of human CCA remained undefined. In the present study, the protein–chemotherapy drug interaction network analysis together with CB-Dock analysis revealed that CIAPIN1 interacts directly with doxorubicin (Figure 11). Doxorubicin is routinely used for the treatment of several cancers [41] and a combined treatment of doxorubicin with salinomycin has been used to enhance doxorubicin sensitivity in CCA patients [42]. In addition, our data indicated that CIAPIN1 interacted indirectly with cisplatin via NOS1 (Figure 11a). Related to this finding, Zou et al. suggested that NOS1 promoted chemoresistance against cisplatin (DDP) in ovarian cancer cells, providing a potential target to reduce chemoresistance to ovarian cancer therapeutics [36]. Thus, we speculate that CIAPIN1 may play an important role in chemoresistance against drugs such as enhancing doxorubicin resistance, and CIAPIN1 may be a novel molecular target for CCA chemotherapy. Further in vitro studies are required to validate the role of CIAPIN1 in the chemoresistance of CCA cells.

## 5. Conclusions

In conclusion, this study revealed that the high expression of CIAPIN1 in CCA tissues is associated with lymph node metastasis and poor survival of CCA patients. CIAPIN1 expression in CCA tissues can serve as a prognostic biomarker for overall survival time and can be an indicator for metastasis of CCA patients. In addition, CIAPIN1 gene silencing caused suppression of CCA cell motilities, suggesting an important role of CIAPIN1 in CCA metastasis. In addition, using bioinformatic analysis, we predicted that the signaling pathway of CIAPIN1 involves the TGF-β/SMAD signaling pathway via NOS1 in CCA cells. Moreover, CIAPIN1 may directly bind to doxorubicin to cause drug resistance. CIAPIN1 might serve as a novel promising molecular target for CCA chemotherapy.

## Figures and Tables

**Table 1:** Clinicopathological associations of CIAPIN1 expression in patients with CCA.

Patients’ Characteristics	CIAPIN1 Expression (H-Score)		*p*
	Low ≤ 207.5(*n* = 15)	High > 207.5(*n* = 24)	
Age (years)	56.67 ± 11.23	61.67 ± 6.53	0.091 ^a^
Gender			0.317 ^b^
Male	11 (73.3%)	13 (54.2%)	
Female	4 (26.7%)	11 (45.8%)	
Tumor size			0.176 ^b,c^
<5 cm	4 (26.7%)	12 (54.5%)	
≥5 cm	11 (73.3%)	10 (45.5%)	
Histopathological grade			0.055 ^b^
Non-Papillary	10 (66.7%)	8 (33.3%)	
Papillary	5 (33.3%)	16 (66.7%)	
Lymph node metastasis			0.010 ^b,^*
No	11 (73.3%)	7 (29.1%)	
Yes	4 (26.7%)	17 (70.9%)	
Intraductal invasion			0.048 ^b,^*
No	11 (73.3%)	9 (37.5%)	
Yes	4 (26.7%)	15 (62.5%)	
Vascular invasion			0.711 ^b^
No	12 (80.0%)	17 (70.8%)	
Yes	3 (20.0%)	7 (29.2%)	
Survival time (days)	562 ± 428	291 ± 225	0.013 ^a,^*

The difference between high- and low-CIAPIN1 groups was estimated using ^a^ Mann–Whitney U test and ^b^ Fisher’s exact test; ^c^ *n* = 37; * Statistically significant correlation as *p* < 0.05.

**Table 2:** Univariate and multivariate Cox regression analyses of clinicopathological parameters and CIAPIN1 protein in CCA tissues.

Clinicopathological Factors	Univariate Analysis				Multivariate Analysis			
	*n*	HR	95% CI	*p*	*n*	HR	95% CI	*p*
Age (years, median range)				0.551				0.740
≤60	21	1.00	-		20	1.00	-	
>60	18	1.31	0.541–3.160		17	0.84	0.307–2.316	
Gender				0.937				0.567
Male	24	1.00	-		23	1.00	-	
Female	15	1.04	0.441–2.426		14	0.73	0.248–2.146	
Tumor size				0.466				0.453
<5 cm	16	1.00	-		16	1.00	-	
≥5 cm	21	1.40	0.565–3.481		21	1.57	0.481–5.150	
Histopathological grade				0.795				0.317
Non-Papillary	18	1.00	-		17	1.00	-	
Papillary	21	1.12	0.469–2.686		20	2.68	0.389–18.422	
Lymph node metastasis				0.217				0.280
No	18	1.00	-		16	1.00	-	
Yes	21	1.73	0.724–4.130		21	1.94	0.583–6.420	
Intraductal invasion				0.822				0.242
No	20	1.00	-		19	1.00	-	
Yes	19	0.91	0.381–2.150		18	0.24	0.033–1.773	
Vascular invasion				0.882				0.847
No	29	1.00	-		28	1.00	-	
Yes	10	1.08	0.392–2.977		9	0.89	0.281–2.834	
CIAPIN1 (H-score)				0.022 *				0.031 *
Low (≤207.5)	15	1.00	-		15	1.00	-	
High (>207.5)	24	3.33	1.187–9.340		22	4.01	1.131–14.195	

HR = hazard ratio; CI = confidence interval. * Statistically significant as *p* < 0.05.

**Table 3:** The KEGG pathway enrichment analysis of CIAPIN1-related proteins in the top 30% of scramble-treated CCA cell line samples by DAVID ver. 6.8.

IDs	Pathway Description	*p*	Count	CIAPIN1-Related Matching Proteins
hsa04020	Calcium signaling pathway	0.0071	6	ERBB3, ITPR1, CACNA1A, ADRA1D, NOS1, ADRA1A
hsa04970	Salivary secretion	0.0206	4	ITPR1, ADRA1D, NOS1, ADRA1A
hsa04730	Long-term depression	0.0630	3	ITPR1, CACNA1A, NOS1
hsa04520	Adherens junction	0.0844	3	SMAD2, CSNK2A1, PARD3

ERBB3 (Receptor Tyrosine-protein Kinase erbB-3), ITPR1 (Inositol 1,4,5-trisphosphate Receptor Type 1), CACNA1A (Voltage-dependent P/Q-type Calcium Channel Subunit Alpha-1A), ADRA1D (Alpha-1D Adrenergic Receptor), NOS1 (Nitric Oxide Synthase, Brain), ADRA1A (Alpha-1A Adrenergic Receptor), SMAD2 (Mothers Against Decapentaplegic Homolog 2), CSNK2A1 (Casein Kinase II Subunit Alpha), and PARD3 (Partitioning defective 3 homolog). EASE score (*p*-value) < 0.1 as default was obtained from DAVID ver. 6.8.

## Data Availability

Not applicable.

## Supplementary Materials

The following supporting information can be downloaded at: https://www.mdpi.com/article/10.3390/jcm11133826/s1, Figure S1: Cut-off value of H-score of CIAPIN1 expression in tissues for the prognosis of CCA as calculated using X-tile; Table S1: The relative expression level of 150 distinct proteins found in the scramble of CCA cell lines.
