# Supplementary material for: Prediction of CIAPIN1 (Cytokine-Induced Apoptosis Inhibitor 1) Signaling Pathway and Its Role in Cholangiocarcinoma Metastasis
Source: J Clin Med. 2022 Jul 1;11(13):3826. doi: 10.3390/jcm11133826 (PMC9267148; doi:10.3390/jcm11133826)
Supplement: Supplementary file 1 [file jcm-11-03826-s001.zip › jcm-1777335-supplementary.pdf]

Supplementary materials:

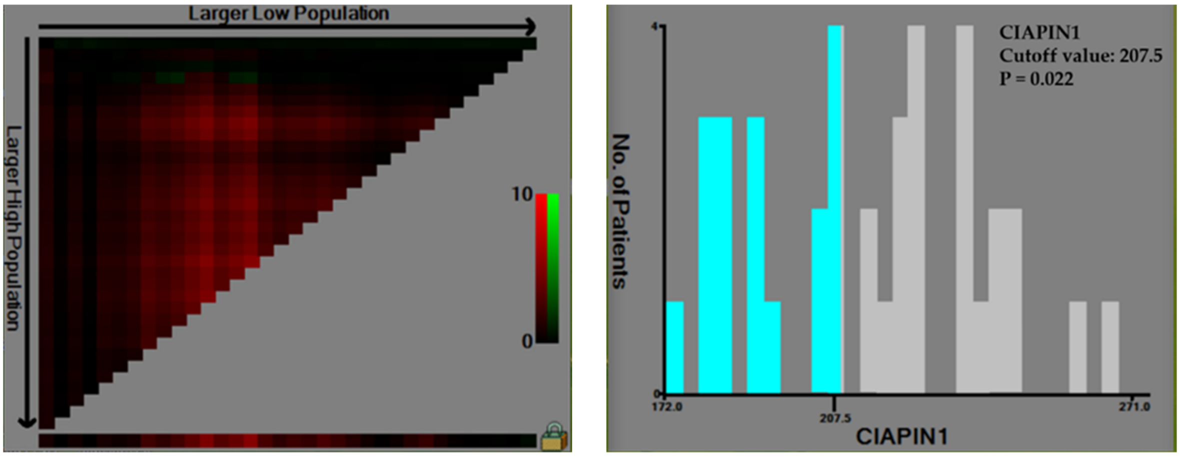

(a) (b)

Figure S1. Cut-off value of H score of CIAPIN1 expression in tissues for the prognosis of CCA as calculated using X-tile. To determine the "optimal" cut-point, our data, including time (survival time (days), censor (alive/dead), and H-score of CIAPIN1 were input into the X-tile. When the cursor is on the X-tile plot, press the left mouse button, and the cursor will move to the point on the plot with the highest value for the currently displayed X-tile plot type. Once there, the plot will lock (a lock icon will appear in the lower right-hand corner of the X-tile window, Figure S1a). You can find the optimal cut-point on the 2-population X-tile plot, Figure S1b.

Table S1. The relative expression level of 150 distinct proteins found in the scramble of CCA cell lines.

**Protein names**	**Protein IDs**	**The log2 fold change of precursor signal intensities**	
		**Scramble KKU-213A[a]**	**Scramble KKU-100[b]**
Serine/threonine-protein phosphatase (EC 3.1.3.16) (Fragment)	Q9BPW0	17.0075529	20.4537248
Cytoplasmic dynein 1 light intermediate chain 2 (Dynein light intermediate chain 2, cytosolic) (LIC-2) (LIC53/55)	O43237	17.0062384	20.1948434
GPALPP motifs-containing protein 1	A0A087WW86	19.7953643	16.857325
Talin-1	Q9Y490	19.6409121	14.9341055
Zinc finger protein 687	Q8N1G0	19.492616	13.3036381
Phosphatidylcholine: ceramide cholinephosphotransferase 2 (EC 2.7.8.27) (Sphingomyelin synthase 2)	Q8NHU3	16.0434546	19.3086368
Mothers against decapentaplegic homolog 2 (Fragment)	K7ERC7	16.096674	19.1910399
Tubulin polyglutamylase TTLL13P	A0A286YFC2	19.0895177	14.8991209
KIAA0427, isoform CRA_a	A0A024R259	12.4777583	18.9726994
Zinc finger RNA-binding protein 2	Q9UPR6	18.9444947	14.5643877
Receptor protein-tyrosine kinase (EC 2.7.10.1)	A0A024RB84	18.6760053	11.4086482
HCG37272, isoform CRA_b	A0A024RA82	18.6557124	12.6165488
Pleckstrin homology-like domain, family B, member 1, isoform CRA_c	A0A024R3F4	18.6002323	13.9955905
Lysine-specific demethylase 3B (Fragment)	H0Y9V5	18.5604075	10.2214663
Protein ECT2 (Epithelial cell-transforming sequence 2 oncogene)	Q9H8V3	13.720458	18.5246641

Collagen alpha-1(XIX) chain (Collagen alpha-1(Y) chain)	Q14993	14.5692623	18.3461155
FLJ00385 protein (Fragment)	Q8NF17	12.6606645	18.2642135
Zinc finger protein 532	Q9HCE3	16.180492	18.2480387
Essential for reactive oxygen species protein	J3KTI1	18.2465087	14.1846429
Clathrin light chain	F8WF69	13.1207239	18.2086726
NK cell receptor D (Fragment)	Q1HEA1	12.4961048	18.1808804
Angiopoietin-related protein 1 (Angiopoietin-3) (ANG-3) (Angiopoietin-like protein 1)	O95841	13.6158595	18.1067674
ATP-binding cassette sub-family B member 10, mitochondrial (ATP-binding cassette transporter 10) (ABC transporter 10 protein) (Mitochondrial ATP-binding cassette 2) (M-ABC2)	Q9NRK6	18.0713786	11.9711483
Retinoic acid-induced protein 1	Q7Z5J4	18.0489977	17.0804636
RNA-binding motif protein, Y chromosome, family 1-member C	P0DJD4	18.0055807	13.9892171
NEDD8-conjugating enzyme UBE2F (Ubiquitin-conjugating enzyme E2F (Putative), isoform CRA_f)	G5E9R6	16.3046722	18.0011883
ER degradation-enhancing alpha-mannosidase-like protein 3 (EC 3.2.1.113) (Alpha-1,2-mannosidase EDEM3)	Q9BZQ6	12.1350679	17.9549233
Taste receptor type 2 member 42 (T2R42) (Taste receptor type 2 member 55) (T2R55)	Q7RTR8	17.9099985	16.5195595
C4a anaphylatoxin (Complement C4 gamma chain)	F5GXS0	17.8745972	16.9299518
Replication initiator 1	C9J590	17.861935	16.4554557
Ubiquitin-protein ligase E3C (EC 2.3.2.26) (HECT-type ubiquitin transferase E3C) (HectH2)	Q15386	17.7704316	16.055007
60S ribosomal protein L8 (Ribosomal protein L8, isoform CRA_a)	G3V1A1	17.7549266	16.7379339
Bifunctional apoptosis regulator (RING finger protein 47)	Q9NZS9	17.7507462	15.4222291
Histone acetyltransferase (EC 2.3.1.48) (Fragment)	A5PKX7	17.7327769	14.3911764
CEBPD protein	Q504X4	17.1915219	17.6898206
Probable E3 ubiquitin-protein ligase HECTD4	F8VWT9	17.628061	16.1736967
Voltage-dependent P/Q-type calcium channel subunit alpha-1A (Brain calcium channel I) (BI) (Calcium channel, L type, alpha-1 polypeptide isoform 4) (Voltage-gated calcium channel subunit alpha Cav2.1)	O00555	16.3290079	17.6210645
Choline transporter-like protein 3 (Solute carrier family 44 member 3)	Q8N4M1	11.6142497	17.6209214
Trichoplein keratin filament-binding protein	A0A024RBM9	17.6178405	16.5303911
Ubiquitin-conjugating enzyme E2 U (Fragment)	V9GZ31	17.6084859	15.898129
Sulfate transporter (Diastrophic dysplasia protein) (Solute carrier family 26 member 2)	P50443	17.5989975	15.7342862
Importin-8 (Fragment)	F5H244	17.5844634	15.5305283
Tyrosine-protein phosphatase non-receptor type 11 (Fragment)	A0A0U1RRI0	12.0399472	17.5472109
FTSJ2 protein (Fragment)	Q1WWK4	10.2726298	17.5135271
Lamin-B2	Q03252	14.0929223	17.4860494
Golgi integral membrane protein 4	F8W785	17.4783272	13.5032046
PH and SEC7 domain-containing protein 3	E5RJ29	14.7922311	17.4716752
Potassium voltage-gated channel subfamily S member 3 (Delayed-rectifier K (+) channel alpha subunit 3) (Voltage-gated potassium channel subunit Kv9.3)	Q9BQ31	17.4581983	12.4934802
Zinc finger protein 2 homolog (Zfp-2) (Zinc finger protein 751)	Q6ZN57	17.4477451	17.4373782
Coiled-coil domain-containing protein 67 (Deuterosome assembly protein 1)	A0A5H1ZRS0	17.4400581	13.3360881

RAR-related orphan nuclear receptor variant 2	F1D8P6	17.4400581	15.8619835
Terminal uridylyltransferase 4	A0A0C4DFM7	11.6022349	17.4049087
p53-induced death domain-containing protein 1 (EC 3.4.21.-) (Leucine-rich repeat and death domain-containing protein) [Cleaved into: PIDD-N; PIDD-C; PIDD-CC]	Q9HB75	17.4049087	14.8908817
Golgin subfamily A member 6-like protein 2	F8WBV9	10.2957689	17.3778036
Target of Nesh-SH3	D3YTG3	16.6115148	17.342474
7-dehydrocholesterol reductase (Fragment)	H0YCS7	16.7863462	17.3228964
Protein phosphatase 1L (EC 3.1.3.16) (Protein phosphatase 1-like) (Protein phosphatase 2C isoform epsilon) (PP2C-epsilon)	Q5SGD2	17.2674634	16.7222739
PH domain leucine-rich repeat-containing protein phosphatase 2	H3BMS5	15.1851852	17.243862
Collectin-12 (Collectin placenta protein 1) (CL-P1) (hCL-P1) (Nurse cell scavenger receptor 2) (Scavenger receptor class A member 4) (Scavenger receptor with C-type lectin)	Q5KU26	12.9021867	17.2435831
39S ribosomal protein L15, mitochondrial (Fragment)	E5RHF4	17.2323835	17.097847
Cysteine and glycine-rich protein 1	E9PP21	10.0887882	17.2287248
PH-interacting protein (PHIP) (DDB1- and CUL4-associated factor 14) (IRS-1 PH domain-binding protein) (WD repeat-containing protein 11)	Q8WWQ0	17.224209	13.5791981
Odorant-binding protein 2b	C9J395	17.1860175	10.5527651
Golgin subfamily A member 6B	A6NDN3	17.1788399	15.2709523
TBC1 domain family member 1 (Fragment)	H0Y8P0	14.6548598	17.1760173
Nitric oxide synthase (EC 1.14.13.39)	C9J5P6	17.1677121	14.8420065
Dynamin-like 120 kDa protein, mitochondrial (EC 3.6.5.5) (Optic atrophy protein 1) [Cleaved into: Dynamin-like 120 kDa protein, form S1]	O60313	17.1541259	12.5975405
NACHT, LRR and PYD domains-containing protein 6 (Angiotensin II/vasopressin receptor) (PYRIN-containing APAF1-like protein 5)	P59044	17.1345065	16.1209466
Inositol 1,4,5-trisphosphate receptor type 1	A0A3B3ITU8	17.1333028	14.7277078
ACACB protein	Q4G170	17.1169531	11.9950251
Myomesin-3 (Myomesin family member 3)	Q5VTT5	17.1154298	16.8968763
Regenerating islet-derived protein 4 (REG-4) (Gastrointestinal secretory protein) (REG-like protein) (Regenerating islet-derived protein IV) (Reg IV)	Q9BYZ8	17.1055403	16.8228944
Dead end protein homolog 1 (RNA-binding motif, single-stranded-interacting protein 4)	Q8IYX4	17.1023653	15.312883
PAX3- and PAX7-binding protein 1 (GC-rich sequence DNA-binding factor 1)	Q9Y5B6	17.0936239	14.7802322
Golgin subfamily A member 3 (Golgi complex-associated protein of 170 kDa) (GCP170) (Golgin-160)	Q08378	17.090526	14.5903531
Tektin-4	Q8WW24	17.0862814	12.9140113
La-related protein 6 (Acheron) (Achn) (La ribonucleoprotein domain family member 6)	Q9BRS8	17.0816083	15.2668231
Voltage-dependent calcium channel beta 2g subunit	Q5UM67	17.0786929	10.9070409
Partitioning defective 3 homolog	Q5VWV2	16.3394151	17.0639217
Zinc finger protein 74 (Zinc finger protein 520) (hZNF7)	Q16587	17.0465485	12.5153549
Protein sprouty homolog 1 (Spry-1)	O43609	16.4750367	17.0345626
Pleckstrin homology domain-containing family H member 1 (PH domain-containing family H member 1)	Q9ULM0	17.0142167	15.9749075
Epididymis secretory protein Li 100	V9HW39	11.4273128	17.008538

Zinc finger protein 117	A0A087X2A5	11.9996477	16.9882631
A6(IV) collagen (Fragment)	Q9UEH6	14.8390069	16.974437
MAP7 domain-containing protein 3 (Fragment)	A0A0A0MRP0	16.9692741	12.0899139
GRIP and coiled-coil domain containing 2, isoform CRA_a	D3DX70	16.9664604	15.0996355
Rho guanine nucleotide exchange factor 17 (164 kDa Rho-specific guanine-nucleotide exchange factor) (p164-RhoGEF) (p164RhoGEF) (Tumor endothelial marker 4)	Q96PE2	16.9579862	13.4937304
Uncharacterized protein DKFZp686J22257	Q68D94	16.9541281	14.7088683
Tetratricopeptide repeat protein 6	G3V3A5	12.4354098	16.9429497
Dynein heavy chain 5, axonemal (Axonemal beta dynein heavy chain 5) (Ciliary dynein heavy chain 5)	Q8TE73	16.9404283	14.0672663
Alpha-1D adrenergic receptor (Alpha-1A adrenergic receptor) (Alpha-1D adrenoreceptor) (Alpha-1D adrenoceptor) (Alpha-adrenergic receptor 1a)	P25100	15.6556425	16.9302984
Teneurin-3 (Ten-3) (Protein Odd Oz/ten-m homolog 3) (Tenascin-M3) (Ten-m3) (Teneurin transmembrane protein 3)	Q9P273	12.4645458	16.9299518
Cadherin EGF LAG seven-pass G-type receptor 1 (Cadherin family member 9) (Flamingo homolog 2) (hFmi2)	Q9NYQ6	15.6117453	16.9207948
KIAA0921 splice variant 1 (Fragment)	Q5W9F7	16.9206785	11.1716772
Alternative protein LOC100129699	L8EBI5	16.9177683	15.3485207
Cadherin EGF LAG seven-pass G-type receptor 3 (Cadherin family member 11) (Epidermal growth factor-like protein 1) (EGF-like protein 1) (Flamingo homolog 1) (hFmi1) (Multiple epidermal growth factor-like domains protein 2) (Multiple EGF-like domains protein 2)	Q9NYQ7	13.3912436	16.9113452
Pleckstrin homology domain-containing family G member 7 (PH domain-containing family G member 7)	Q6ZR37	16.9100571	12.430348
Bystin	Q13895	16.3863509	16.906186
Helicase SRCAP	A0A0A0MS59	16.9035995	11.1761731
Reelin	J3KQ66	16.8711111	15.7368511
Transcription factor 4	A0A1B0GW91	12.7148894	16.8661681
IKIP protein (Fragment)	Q3B7K3	16.6949291	16.8530658
Sialic acid-binding Ig-like lectin 6 (Siglec-6) (CD33 antigen-like 1) (CDw327) (Obesity-binding protein 1) (OB-BP1) (CD antigen CD327)	O43699	16.8286076	12.3698427
Dedicator of cytokinesis protein 3 (Modifier of cell adhesion) (Presenilin-binding protein) (PBP)	Q8IZD9	15.4540743	16.8097681
Chromodomain-helicase-DNA-binding protein 1 (CHD-1) (EC 3.6.4.12) (ATP-dependent helicase CHD1)	O14646	12.7442032	16.7811035
Centrosomal protein of 290 kDa (Cep290) (Bardet-Biedl syndrome 14 protein) (Cancer/testis antigen 87) (CT87) (Nephrocystin-6) (Tumor antigen se2-2)	O15078	16.3395717	16.7579889
Little elongation complex subunit 1 (Interactor of little elongator complex ELL subunit 1)	Q9Y2F5	13.9551958	16.7502883
Centrosomal protein of 57 kDa (Fragment)	F5H1B0	16.7260052	12.6696823
Heterogeneous nuclear ribonucleoprotein A/B	D6R9P3	16.7256059	13.4641628
WSC domain-containing protein 2	Q2TBF2	15.829475	16.7204046
BOP1 protein (Fragment)	Q4G0D9	16.706577	11.3238429
Casein kinase II subunit alpha (Fragment)	A0A2R8YEW1	14.8306637	16.7036062

Brain-specific angiogenesis inhibitor 1-associated protein 2-like protein 1 (BAI1-associated protein 2-like protein 1) (Insulin receptor tyrosine kinase substrate)	Q9UHR4	16.7007648	13.6565366
Coagulation factor VIII (Antihemophilic factor) (AHF) (Procoagulant component) [Cleaved into: Factor VIIIa heavy chain, 200 kDa isoform; Factor VIIIa heavy chain, 92 kDa isoform; Factor VIII B chain; Factor VIIIa light chain]	P00451	16.6998164	12.4767715
tRNA (guanine(37)-N1)-methyltransferase (EC 2.1.1.228) (M1G-methyltransferase) (tRNA [GM37] methyltransferase) (tRNA methyltransferase 5 homolog)	Q32P41	16.6896159	14.6318023
Zinc finger protein 564	C9JLM4	14.2514084	16.6655302
Vang-like protein 2 (Loop-tail protein 1 homolog) (Strabismus 1) (Van Gogh-like protein 2)	Q9ULK5	12.9745938	16.6616389
Protein shisa-6	Q6ZSJ9	11.1686721	16.6153997
Trafficking protein particle complex subunit 2-like protein	H3BQQ8	16.6110824	16.0138897
Spermatogenesis-associated protein 31D4 (Protein FAM75D4)	Q6ZUB0	16.5993172	14.8993569
Neurofibromin 2 (Bilateral acoustic neuroma), isoform CRA_i	A0A024R1F6	16.5690991	10.0575857
Probable helicase with zinc finger domain (EC 3.6.4.-) (Down-regulated in human cancers protein)	P42694	14.8694011	16.5540342
MMS19 nucleotide excision repair protein homolog (hMMS19) (MET18 homolog) (MMS19-like protein)	Q96T76	16.2515009	16.5333145
Zinc finger protein AEBP2 (Adipocyte enhancer-binding protein 2) (AE-binding protein 2)	Q6ZN18	16.5105324	11.2247253
Uncharacterized protein FP972	Q71MF7	12.3108967	16.5017595
Alternative protein HSPC159	L0R6H9	15.9838843	16.4973997
Protein SMG7 (EST1-like protein C) (SMG-7 homolog) (hSMG-7)	Q92540	16.4886561	15.2000557
Somatostatin receptor type 4 (SS-4-R) (SS4-R) (SS4R)	P31391	12.3525952	16.4691966
Alternative protein NBPF9	L8E7E2	16.4691171	16.1203796
Transcription factor RFX4 (Fragment)	R4GMS3	16.4681785	16.1312742
Interferon alpha-4 (IFN-alpha-4) (Interferon alpha-4B) (Interferon alpha-76) (Interferon alpha-M1)	P05014	16.4460494	13.1295567
SH2B adapter protein 2 (Adapter protein with pleckstrin homology and Src homology 2 domains) (SH2 and PH domain-containing adapter protein APS)	O14492	16.4448531	10.4692348
Acyl-Coenzyme A dehydrogenase family, member 8, isoform CRA_b (cDNA FLJ43383 fis, clone OCBBF2006058, highly similar to Homo sapiens acyl-Coenzyme A dehydrogenase-8 mRNA)	Q6ZUS0	16.4437044	14.0215005
Zinc finger protein 710	Q8N1W2	13.6045532	16.4190385
Septin-4	A0A5F9ZHH3	15.5725522	16.3890935
Beta-galactoside alpha-2,6-sialyltransferase 1 (Fragment)	H7C472	16.3863846	15.4185444
General transcription factor 3C polypeptide 4 (EC 2.3.1.48) (TF3C-delta) (Transcription factor IIIC 90 kDa subunit) (TFIIIC 90 kDa subunit) (TFIIIC90) (Transcription factor IIIC subunit delta)	Q9UKN8	14.850724	16.3827929
KIF5B-RET(NM_020630) _K22R12 fusion protein	M1V490	15.2493727	16.3673806
Putative cleavage and polyadenylation-specificity factor subunit 4-like protein	H9KVA5	15.7701477	16.3637754
MYO1E variant protein (Myosin IE) (cDNA FLJ76307, highly similar to Homo sapiens myosin IE (MYO1E), mRNA)	Q4KMR3	12.5264992	16.3612413

Neurofilament heavy polypeptide (NF-H) (200 kDa neurofilament protein) (Neurofilament triplet H protein)	P12036	15.0137152	16.3568646
Golgi apparatus protein 1 (CFR-1) (Cysteine-rich fibroblast growth factor receptor) (E-selectin ligand 1) (ESL-1) (Golgi sialoglycoprotein MG-160)	Q92896	16.2942797	12.9379255
Uncharacterized protein DKFZp686D17136 (Fragment)	Q68CN5	16.288091	12.2256895
R-spondin-1 (Roof plate-specific spondin-1) (hRspo1)	Q2MKA7	16.2813686	10.9576825
Sorting nexin-14	D6RDH9	14.8212259	16.2224176
Chromodomain-helicase-DNA-binding protein 5 (CHD-5) (EC 3.6.4.12) (ATP-dependent helicase CHD5)	Q8TDI0	16.2182416	15.8199296
Vacuolar fusion protein MON1 homolog A	Q86VX9	16.2179956	15.1557274
StAR-related lipid transfer protein 9 (START domain-containing protein 9) (StARD9)	Q9P2P6	14.2675914	16.2149262
Activating signal cointegrator 1 complex subunit 1 (ASC-1 complex subunit p50) (Trip4 complex subunit p50)	Q8N9N2	12.8079586	16.2064802

[a] Values represent the log2 fold change of precursor signal intensities in particular scramble KKU-213A compared to siRNA KKU-213A detected by LC-MS/MS; [b] Values represent the log2 fold change of precursor signal intensities in particular scramble KKU-100 compared to siRNA KKU-100 detected by LC-MS/MS
